# Genome scale patterns of supercoiling in a bacterial chromosome

**DOI:** 10.1038/ncomms11055

**Published:** 2016-03-30

**Authors:** Avantika Lal, Amlanjyoti Dhar, Andrei Trostel, Fedor Kouzine, Aswin S. N. Seshasayee, Sankar Adhya

**Affiliations:** 1 National Centre for Biological Sciences, Tata Institute of Fundamental Research, Bangalore 560065, Karnataka, India; 2 Laboratory of Molecular Biology, Center for Cancer Research, National Cancer Institute, National Institutes of Health, Bethesda, Maryland 20892-4255, USA; 3 Laboratory of Pathology, National Cancer Institute, National Institutes of Health, Bethesda, Maryland 20892-4255, USA

## Abstract

DNA in bacterial cells primarily exists in a negatively supercoiled state. The extent of supercoiling differs between regions of the chromosome, changes in response to external conditions and regulates gene expression. Here we report the use of trimethylpsoralen intercalation to map the extent of supercoiling across the *Escherichia coli* chromosome during exponential and stationary growth phases. We find that stationary phase *E. coli* cells display a gradient of negative supercoiling, with the terminus being more negatively supercoiled than the origin of replication, and that such a gradient is absent in exponentially growing cells. This stationary phase pattern is correlated with the binding of the nucleoid-associated protein HU, and we show that it is lost in an HU deletion strain. We suggest that HU establishes higher supercoiling near the terminus of the chromosome during stationary phase, whereas during exponential growth DNA gyrase and/or transcription equalizes supercoiling across the chromosome.

## Abstract (web summary)

Bacterial DNA primarily exists in a negatively supercoiled or under-wound state. Here, by mapping the supercoiling state, the authors show that there is a gradient of supercoiling across the bacterial chromosome with the terminus being more negatively supercoiled than the origin.

## Graphical abstract

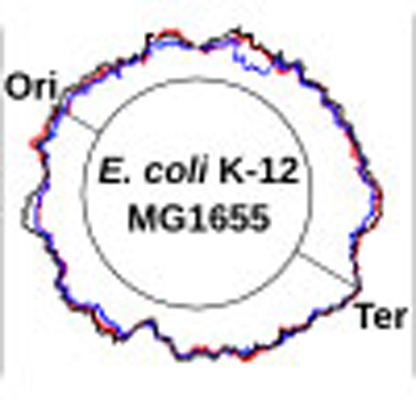

DNA from living cells is mostly negatively supercoiled *in vivo*^1^. This negative supercoiling is important for transcription, replication and recombination^2^,^3^,^4^,^5^,^6^ and is brought about by the combined action of transcription, replication, topoisomerase activity, and the binding of proteins such as bacterial nucleoid-associated proteins (NAPs) to DNA.

During transcription, the moving RNA polymerase creates a region of positive supercoiling (overwinding) in front of itself and negative supercoiling (underwinding) behind^7^,^8^. In *Escherichia coli*, these negative supercoils are relaxed by the action of Topoisomerase I and the positive supercoils are relaxed by ATP-dependent DNA gyrase, so that the balance between the activities of the two enzymes determines the overall level of supercoiling^7^,^8^. This is referred to as unconstrained supercoiling. Supercoiling can also be constrained by nucleoid-associated proteins such as H-NS, HU and FIS. HU is present in ∼30,000 dimers per cell^9^,^10^ and is the most conserved NAP across bacterial species. It binds across the bacterial chromosome and has been shown to constrain negative supercoils on DNA *in vitro*^11^,^12^,^13^. This is in conflict with transposon insertion screens^14^, which identified H-NS and FIS, but not HU or its homologue IHF, as modulators of DNA supercoiling. However, other studies^15^,^16^ have seen reduced supercoiling of both plasmid and chromosomal DNA in HU knockouts. H-NS, a gene silencer that binds in long tracts to A+T-rich and / or intrinsically bent DNA, has been shown to constrain negative supercoils both *in vivo* and *in vitro*^17^,^18^. FIS, the most abundant NAP during exponential growth of *E. coli,* constrains a low superhelical density *in vitro* and also modulates the activity of DNA gyrase^19^,^20^.

The extent of supercoiling is sensitive to changes in the extracellular environment such as nutrient starvation^21^, anaerobic growth^22^, osmotic shock^23^,^24^, and temperature^25^. It also acts as a global regulator of gene expression, with sets of genes showing distinct changes in expression upon changes in supercoiling^26^,^27^. As a result, the superhelical state of the DNA connects environmental changes to gene expression states. One condition where chromosomal supercoiling has been suggested to respond to an environmental change and regulate gene expression is stationary phase. *E. coli* cells enter stationary phase upon exhaustion of nutrients; in this state, transcription, translation and proliferation are reduced and cells become more stress-tolerant. Many of the changes in gene expression underlying this process are attributable to the reduced activity of the housekeeping sigma factor σ^70^ and the increased activity of the alternative sigma factor σ^38^ (ref. 28). Average chromosomal supercoiling decreases during stationary phase^21^,^26^,^29^ and it has been suggested that this contributes to the reduced synthesis of ribosomal RNA^30^ and increased activity of σ^38^ over σ^70^ (ref. 29).

Despite these studies, we do not know whether there exists a global pattern to supercoiling along a bacterial chromosome. Based on ChIP-chip (chromatin immunoprecipitation) studies of DNA gyrase, it has been suggested^31^ that during exponential growth there is a gradient of supercoiling with the origin of replication more negatively supercoiled than the terminus. However, this is an indirect measure of supercoiling, one step away from a direct measure of superhelical density.

Here we address this gap using psoralen crosslinking of chromosomal DNA followed by DNA microarray experiments. We find that the stationary phase *E. coli* chromosome displays a gradient of negative supercoiling, with maximal supercoiling near the terminus, and that this gradient is lost in exponential phase. This gradient is also absent in a strain lacking the nucleoid-associated protein HU. We suggest that HU binding maintains negative supercoiling around the terminus in stationary phase, and that DNA gyrase and / or transcriptional activity near the origin equalize supercoiling across the chromosome in exponential phase.

## Results

### Measuring genome-wide supercoiling with psoralen

Psoralens are furanocoumarin compounds that intercalate between DNA base pairs and form crosslinks with DNA under ultraviolet light^32^,^33^. Psoralen binds preferentially to negatively supercoiled DNA^34^, with the frequency of crosslinking being proportional to the superhelical density of the DNA^35^,^36^. Intercalation of psoralen and its derivatives has therefore been used as a measure of average supercoiling of *E. coli* plasmids and genomic DNA^36^, and supercoiling near specific genes^37^. More recently, it has been used to measure local supercoiling across the genome in yeast^35^ and human cells^38^,^39^. It is to be noted here that psoralen crosslinking measures the contribution of twist, but not writhe, to the degree of supercoiling.

We grew *E. coli* cells to mid-exponential or stationary phase in LB medium (growth curves in Supplementary Figure 1), treated them with trimethylpsoralen (hereafter referred to simply as psoralen) and exposed them to UV light. Under these conditions, psoralen enters cells, intercalates between DNA base pairs, and crosslinks the two strands of DNA at a rate proportional to the local superhelical density^35^,^36^,^38^.

Following this principle, we standardized a method to measure the extent of psoralen crosslinking along the *E. coli* chromosome by fragmenting DNA and hybridizing crosslinked and non-crosslinked DNA fragments separately to high-resolution tiling microarrays that cover the entire *E. coli* genome. Since crosslinking by psoralen increases with local supercoiling, we expected that the more negatively supercoiled a given portion of the genome was at the time of psoralen treatment, the more it would be enriched in the crosslinked fraction relative to the non-crosslinked fraction. We calculated this enrichment in the form of the log_2_ ratio of the crosslinked and the non-crosslinked fluorescent signals corresponding to each probe on the microarray. The enrichment ratios were represented as a function of the position on the chromosome to which the corresponding probe mapped. The noisy nature of the data necessitated smoothing of the signal, and the degree of smoothing was as small as possible to provide replicate correlations of 0.75 or greater (Supplementary Figure 2). The smoothed and log-transformed ratio between the signals from the crosslinked and the non-crosslinked fractions was taken as a measure of psoralen binding and thus of local negative supercoiling^38^.

### A terminus centric supercoiling gradient in stationary phase

**Figure 1:**
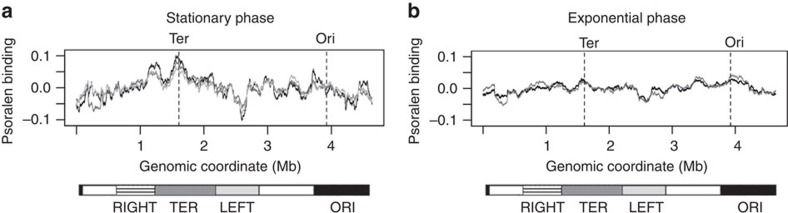
Supercoiling patterns across the E. coli chromosome. (**a**) Psoralen binding in stationary phase. Black, dark grey and light grey tracks represent three independent replicates. The moving average of the signal is plotted with a window size of 200 Kb. Dashed lines mark the positions of the terminus and origin of replication. Lower bars represent chromosomal macrodomains^54^. (**b**) Psoralen binding in mid-exponential phase. Black and grey tracks represent two independent replicates. Dashed lines mark the positions of the terminus and origin of replication. Lower bars represent chromosomal macrodomains.

In stationary phase, we observe that negative supercoiling is greatest close to the terminus of the chromosome and decreases in either direction (Fig. 1a). The range of psoralen binding is from −0.1 to 0.1, consistent with similar data from human chromosomes^38^. Earlier experiments^35^,^36^ have shown that DNA with high physiological levels of negative supercoiling has only about twofold higher psoralen intercalation than relaxed DNA. The terminus region of the chromosome has higher A+T content, and psoralen is known to have a preference for binding to A+T-rich DNA^35^. However, the gradient of supercoiling during stationary phase remains when the signal is corrected for the A+T content of the microarray probes (Supplementary Figure 3), indicating that the observed higher psoralen binding around the terminus is not merely an artifact due to its preference for the higher A+T content in this region. In exponential phase, the level of negative supercoiling is similar across the chromosome, with the origin only slightly more negatively supercoiled than the terminus (Fig. 1b).

### Factors influencing exponential phase supercoiling

**Figure 2:**
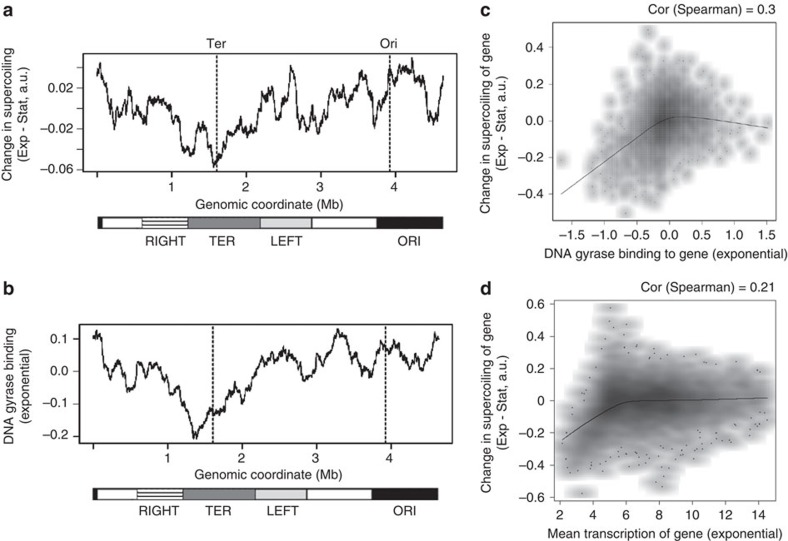
The effect of DNA gyrase binding and transcription on chromosome supercoiling. (**a**) Change in negative supercoiling from stationary to exponential phase. The averages of the psoralen binding signals from both phases were scaled to zero before subtracting the stationary phase signal from the exponential phase signal. As overall negative supercoiling increases during exponential phase, this difference can be assumed to represent the relative increase in supercoiling across the chromosome. (**b**) Binding of DNA gyrase to the *E. coli* chromosome during exponential phase. Both graphs show the moving average of the signal with a window size of 200 Kb. Dashed lines mark the positions of the terminus and origin of replication. Lower bars represent chromosomal macrodomains. (**c**) Smoothed scatter plot of the change in supercoiling from stationary to exponential phase versus DNA gyrase binding within genes in exponential phase. (Permutation test, *P*<10^−5^). Black line represents the loess fit. (**d**) Smoothed scatter plot of change in supercoiling from stationary to exponential phase versus exponential phase transcription level of genes. Black lines represent loess fit.

DNA gyrase introduces negative supercoils into the chromosome by an ATP-dependent mechanism^40^. The activity of DNA gyrase depends on the cellular [ATP]/[ADP] ratio and therefore increases during exponential growth. As a result, the average negative supercoiling of the entire chromosome is higher in exponential phase than in stationary phase^21^,^26^,^29^. However, DNA gyrase does not bind uniformly across the genome in exponential phase; its binding is lowest at the terminus and increases toward the origin^41^. Since the gradient of DNA gyrase binding is opposite to that of stationary phase supercoiling, we reasoned that DNA gyrase might be one of the factors responsible for neutralizing the supercoiling gradient in exponential phase. The difference between the exponential and stationary phase supercoiling levels across the chromosome is plotted in Fig. 2a. Negative supercoiling increases more around the origin than the terminus in exponential phase, and the pattern of this increase is similar to that of DNA gyrase binding across the chromosome^41^ which is plotted in Fig. 2b. A scatter plot of the change in supercoiling from stationary to exponential phase against DNA gyrase binding to each gene (Fig. 2c) illustrates the positive correlation between the two (permutation test, *P*<10^−5^). This suggests that this pattern of increased supercoiling may be created, at least in part, by the action of DNA gyrase.

Previous literature has identified sequence motifs to which DNA gyrase preferentially binds. These include the G+C-rich Repetitive Extragenic Palindromic (REP) sequences^42^, which are present in ∼700 copies in the *E. coli* chromosome. The density of REP sequences is highest near the origin and lowest at the terminus (Supplementary Figure 4a), somewhat similar to the pattern of DNA gyrase binding, and genes near REP sequences show higher DNA gyrase binding (Supplementary Figure 4b, Wilcoxon test *P*<10^−3^).

However, in addition to this, the function of DNA gyrase is tightly linked to transcription. The activity of RNA polymerase generates positive supercoils in front of it, and negative supercoils behind. Topoisomerases release these tensions, with DNA gyrase responsible for releasing the positive supercoils generated in front. In addition, transcription by itself might negatively supercoil DNA in wild-type *E. coli* with functional DNA gyrase and topoisomerase I; this might be explained by the formation of the transcription bubble^43^. We observe that exponential phase gene expression measurements from tiling microarrays are correlated, to a small extent, with the binding profile of DNA gyrase (Supplementary Figure 5), as well as with the difference in psoralen crosslinking between exponential and stationary phases (Fig. 2d). Therefore, it remains unclear whether the changes in supercoiling patterns between the stationary and exponential phases of growth emerge directly from the activity of DNA gyrase itself, or whether it is a function of the combined action of the RNA polymerase and the two topoisomerases. Separating these effects presents a significant challenge.

### The role of HU in maintaining the supercoiling gradient

We next examined the association of negative supercoiling with the binding of prominent NAPs of *E. coli*: H-NS, HU, IHF and FIS. While binding sites of IHF and FIS showed no difference in psoralen binding compared to neighbouring regions of DNA (Supplementary Figure 6; Supplementary Figure 7), we found an association with psoralen binding for HU and H-NS.

**Figure 3:**
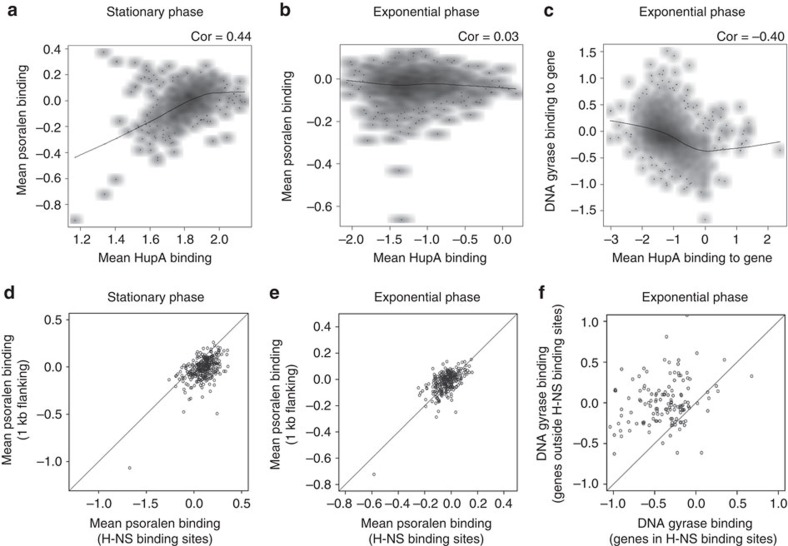
Nucleoid-associated proteins are associated with supercoiling. (**a**) Smoothed scatter plot of average psoralen binding versus average HupA binding in 5 Kb bins covering the entire *E. coli* genome, during stationary phase. Black line represents loess fit. (**b**) Smoothed scatter plot of average psoralen binding versus average HupA binding in 5 Kb bins covering the entire *E. coli* genome, during exponential phase. Black line represents loess fit. (**c**) Smoothed scatter plot of DNA gyrase binding versus average HupA binding to genes in exponential phase. Black line represents loess fit. (**d**) Scatter plot of average psoralen binding in H-NS binding sites versus average psoralen binding in the 1 Kb regions flanking them, during stationary phase (Paired Wilcoxon test *P*<10^−15^). Black line has slope=1. (**e**) Scatter plot of average psoralen binding in H-NS binding sites versus average psoralen binding in the 1 Kb regions flanking them, during exponential phase. Black line has slope=1. (**f**) Scatter plot of DNA gyrase binding in 135 pairs of genes less than 1 Kb apart, where one gene is within an H-NS binding site and the other is outside. Black line has slope=1.

HU binds throughout the *E. coli* chromosome. To study its effects on DNA supercoiling we divided the genome into 5 Kb bins, and calculated the extent of HU binding in each bin. We observed a positive correlation between psoralen binding and the binding of the HupA subunit of HU during stationary phase (Fig. 3a, Pearson correlation coefficient 0.44). Results were similar for the HupB subunit (Supplementary Figure 8). H-NS binding sites had higher psoralen binding (and therefore higher negative supercoiling) compared to 1 Kb long stretches of DNA flanking them on either side, during stationary phase (Fig. 3d, paired Wilcoxon test *P*<10^−15^). Note, however, that during exponential phase, the binding profile of HU shows no correlation with psoralen crosslinking (Fig. 3b), and H-NS binding sites also tend to have lower psoralen binding than their flanking regions (Fig. 3e). One possible explanation could be reduced DNA gyrase binding at HU or H-NS-bound loci (Fig. 3c,f), due to reduced accessibility of the DNA or reduced transcription.

Could HU or H-NS be responsible for the supercoiling gradient in stationary phase? Towards answering this, we first performed a computational analysis, followed by an experimental investigation for HU.

**Figure 4:**
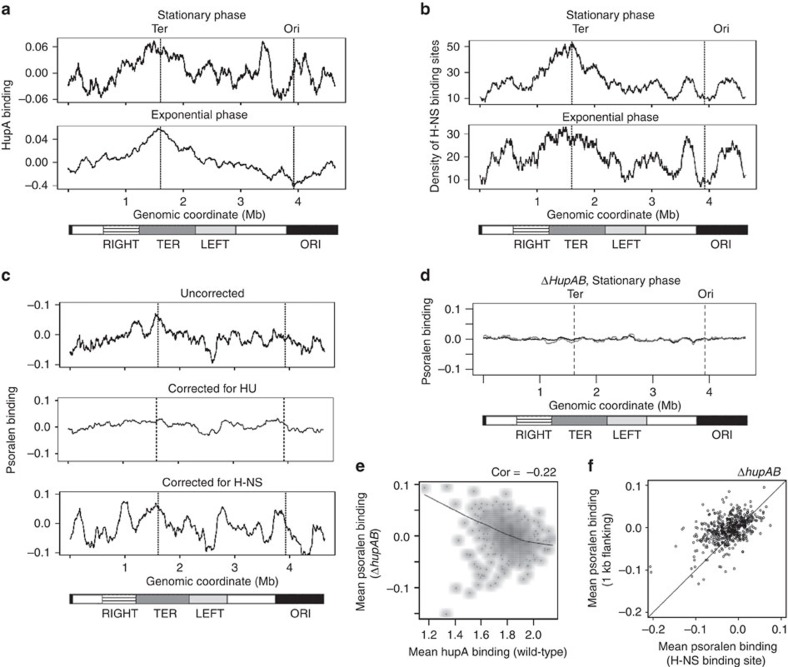
Role of H-NS and HU in maintaining the supercoiling gradient. (**a**) HupA binding across the *E. coli* chromosome during stationary phase (upper) and exponential phase (lower). The moving average of the signal is plotted with a window size of 200 Kb. (**b**) Number of H-NS binding sites in a 200 Kb sliding window across the *E. coli* chromosome during stationary phase (upper) and exponential phase (lower). (**c**) Plot of psoralen binding across the chromosome, uncorrected (upper), corrected for the effect of HU (middle), and corrected for the effect of H-NS (lower). (**d**) Psoralen binding in the HU knockout strain during stationary phase. Black and grey tracks represent two independent replicates. The moving average of the signal is plotted with a window size of 200 Kb. For (**a**–**d**) dashed lines mark the positions of the terminus and origin of replication, and lower bars represent chromosomal macrodomains. (**e**) Smoothed scatter plot of average psoralen binding in the HU knockout versus average HupA binding in the wild-type, in 5 Kb bins covering the entire genome, during stationary phase. Black line represents loess fit. (**f**) Scatter plot of average psoralen binding in H-NS binding regions versus 1 Kb flanking regions, in the HU knockout during stationary phase. Black line has slope=1.

In the computational approach, using the relationship between the binding profiles of HU or H-NS (Fig. 4a,b) and the psoralen binding signal in stationary phase (Fig. 4c upper), we estimated what the supercoiling pattern of the stationary phase chromosome would be in the absence of these proteins. To correct for the effect of HU we divided the genome into 5 Kb bins and plotted the residuals of the loess fit between psoralen binding and HU binding levels in each bin (Fig. 4c middle). To correct for the higher negative supercoiling of H-NS binding sites, we took the psoralen binding in each H-NS binding site plus 2.5 Kb flanking regions on either side, and replaced these values with the average psoralen binding in the 2 Kb regions on either side. The 2.5 Kb flanking region was included as this region also has slightly increased supercoiling, decaying with distance from the H-NS binding site (Supplementary Figure 9). Thus we reduced the psoralen binding signal within and immediately adjacent to the H-NS binding site to that of neighbouring unbound DNA, and plotted the resultant pattern of psoralen binding (Fig. 4c lower). We observed that correcting for the effect of H-NS did not change the basic pattern of supercoiling in stationary phase. Correcting for HU had a considerably larger effect, suggesting that HU may have a larger role in maintaining the pattern of supercoiling during stationary phase.

Given the general overlap in global binding patterns between H-NS and HU, it is entirely possible that at least one of the two correlations is incidental. Psoralen crosslinking experiments measure negative supercoiling manifested by local DNA unwinding; therefore it is not clear how a DNA zipper like H-NS could result in local DNA unwinding^44^. For HU, however, there is evidence that its deletion results in a decrease in global negative superhelicity^45^, at least in exponential phase. A role for HU in unwinding bound DNA has also been shown^46^. Therefore, we experimentally investigated the effect of a HU deletion on the pattern of psoralen crosslinking during stationary phase.

To test experimentally the prediction that HU maintains the gradient of supercoiling around the terminus in stationary phase, we measured psoralen binding across the chromosome in a *ΔhupAB* strain of *E. coli,* which lacks both the α and β subunits of HU. We found that as predicted, the absence of HU resulted in the loss of the peak of supercoiling surrounding the terminus, giving a flat supercoiling profile (Fig. 4d). This is unlikely to be due to changes in transcription in the HU knockout, as there was no particular difference in gene expression around the terminus between the HU knockout and the wild-type, during stationary phase (Supplementary Figure 10).

The positive correlation observed between HU binding and psoralen binding in the wild-type strain was reversed in the HU deletion strain, with regions that were highly HU-bound in the wild-type showing lower psoralen binding in the HU knockout (Fig. 4e, Pearson correlation coefficient −0.22). Further, the trend for H-NS binding sites to have higher psoralen binding than their flanking regions was reversed in the absence of HU (Fig. 4f), suggesting that the connection between H-NS and psoralen binding shown in Fig. 3d was not a direct consequence of H-NS binding to DNA.

Thus the binding of HU leads to higher negative supercoiling of its bound or neighbouring DNA, resulting in overall higher negative supercoiling surrounding the chromosomal terminus during stationary phase.

## Discussion

We have demonstrated that stationary phase *E. coli* cells maintain a gradient of negative supercoiling with its peak near the terminus of the chromosome, whereas exponentially growing *E. coli* show relatively even supercoiling across the chromosome. Previously, Sobetzko *et al.*^31^ have proposed that during exponential phase, DNA gyrase maintains a gradient of supercoiling with the origin higher than the terminus, favoring transcription of origin-proximal genes as well as the initiation of replication at OriC. Though overall negative supercoiling is known to increase in exponential phase, we see very little difference between the origin and other regions of the chromosome.

We propose that during stationary phase, factors such as the nucleoid-associated protein HU maintain a higher level of supercoiling around the terminus. Previous studies have shown that psoralen crosslinking to the DNA is prevented by nucleosomes^38^. Our analysis suggests that HU-bound DNA shows higher psoralen crosslinking; an association which is lost in HU-deficient cells. This appears to be in conflict with the accepted wisdom that psoralen crosslinking typically occurs at unconstrained and unwound DNA. The level to which DNA bound to HU is unwound may favour crosslinking by psoralen, whereas a nucleosome wrapping DNA around it might pose a greater steric challenge for psoralen-DNA interactions. Alternatively, psoralen crosslinking might occur at unwound DNA adjacent to where HU is bound: the present experiments are unlikely to help resolve this.

On entry into exponential phase, the increased [ATP]/[ADP] ratio leads to increased DNA gyrase activity, which together with high transcription (and possibly other factors) brings about higher negative supercoiling. However, the activities of DNA gyrase and RNA polymerase increase with proximity to the origin. Thus, the general increase in negative superhelicity of the chromosome of exponentially growing *E. coli* might be localised to origin-proximal regions.

This is the first study of genome-wide supercoiling patterns in bacteria. Future experiments may further elucidate how these patterns change under different conditions and species. While we have experimentally shown the role of HU in maintaining chromosomal supercoiling in stationary phase, further experiments will evaluate our predictions on the functions of DNA gyrase and transcription. We expect that inhibition of DNA gyrase should restore the terminus-to-origin supercoiling gradient in exponential phase. However, a challenge here would be to decouple the interlinked effects of DNA gyrase and transcription.

## Methods

### Bacterial strain and cell culture

*E. coli* str. K-12 MG1655 cells were grown in LB medium at 37 °C with aeration, until mid-exponential phase (OD_600_=0.95) or stationary phase (OD_600_=2.4).

### Crosslinking with Psoralen

Cells from 25 ml culture were harvested by centrifugation at the given time points and washed with cold phosphate buffer (pH 7.2). The cells were resuspended in 10 ml Tris-Cl buffer (pH 8) and incubated at 37 °C for 2 min. EDTA (final 0.5 mM) was added and cells were incubated at the same temperature for another two minutes. The cells were immediately chilled on ice and MgCl_2_ (final 1 mM) was added. 100 μl of saturated trimethylpsoralen solution in ethanol (Sigma-Aldrich, cat. no T6137) was added to the chilled cell suspension, mixed by gentle shaking and incubated at 4 °C for 10 min. The cells were then exposed to UV light of wavelength 365 nM and intensity 1.2 kJ.m^−2^min^−1^ for 45 s, immediately washed with M69 buffer and finally resuspended in the same buffer. Three biological replicate experiments were performed for wild-type cells in stationary phase and two for all other conditions.

### DNA isolation and fragmentation

Cells were lysed by adding EDTA (final 0.1 M) and 2% SDS and incubating at 37 °C for 1 h followed by addition of 100 μl proteinase K (20 mg ml^−1^ stock) and overnight incubation at the same temperature. After complete cell lysis, genomic DNA was isolated by the phenol-chloroform method, treated overnight with DNase free RNase (Roche, cat. no. 11119915001) and purified by the phenol-chloroform method. DNA was resuspended in tris buffer (pH 8) and sheared by sonication to a median length of 300 bp.

### Separation of crosslinked and non-crosslinked fragments

Sheared DNA was electrophoresced in an agarose gel, eluted, concentrated by ethanol precipitation and dissolved in 25 mM sodium phosphate buffer (pH 7). The DNA was heated at 95 °C for 2 min followed by quick addition of DMSO (final 50%) and glyoxal (final 6–8% by volume) and reheating at 60 °C for two hours. Glyoxal treated DNA fragments were separated on a 3.5% agarose gel in phosphate buffer. The gel was incubated with denaturing solution (0.5 M NaOH, 1.5 M NaCl) at 65 °C for 3 h to reverse psoralen crosslinks. Crosslinked and non-crosslinked DNA fragments were excised separately from the gel, eluted, and concentrated by ethanol precipitation.

### DNA labelling

Fragmented DNA was 3′ termini biotin labelled using the GeneChip DNA Labelling Reagent (Affymetrix 900542) and 60U of Terminal Deoxynucleotidyl Transferase (Promega M1875) at 37 °C for 60 min. The labelling reaction was stopped by the addition of 0.5M EDTA.

### Microarray hybridization

The crosslinked and non-crosslinked DNA was hybridized separately to high-resolution tiling microarrays. Labelled DNA fragments (3 ug) were hybridized for 16 h (60 rpms) at 45 °C to tiling array chips (Ecoli_Tab520346F) purchased from Affymetrix (Santa Clara, CA). The array covers the entire *E. coli* K-12 MG1655 genome with 25 bp long probes at a resolution of 4 bp between steps, and also contains 33,996 control probes against non-*E. coli* genomes.

### Microarray staining and scanning

The chips were washed with Wash Buffer A: Non-Stringent Wash Buffer [6X SSPE, 0.01% Tween-20], Wash Buffer B: [100 mM MES, 0.1 M [Na^+^], 0.01% Tween-20] and stained with Streptavidin Phycoerythrin (Molecular Probes S-866) and anti-streptavidin antibody (goat), biotinylated (Vector Laboratories BA-0500; final 5 μg ml^−1^) on a Genechip Fluidics Station 450 (Affymetrix) according to washing and staining protocol, ProkGE-WS2_450. Hybridized, washed and stained microarrays were scanned using a Genechip Scanner 3,000 (Affymetrix).

### Microarray data analysis

Probe intensities were corrected for background by subtracting the median intensities of control (non-*E. coli*) probes having the same G+C content. For psoralen binding experiments, this was followed by quantile normalization of signals from all arrays and log_2_ transformation. For each probe, log-transformed signals from the non-crosslinked DNA samples were subtracted from those of the corresponding crosslinked DNA samples. The resultant values of log_2_(crosslinked signal/non-crosslinked signal) were taken as a measure of psoralen binding to each sequence. To obtain an average psoralen binding signal, signals were smoothed using a moving average with a window size of 1.1 Kb and averaged between replicates. Smoothed signals showed a correlation of 0.75–0.87 between replicates. All analyses were carried out in R version 3.1.0. Raw microarray data was analysed using the Starr 1.18.1 package^47^. Nonparametric regression was done using the lowess smoothing function in R.

### External data sources

ChIP-chip data for DNA gyrase binding to genes in exponential phase was taken from Jeong *et al.* (2004)^41^. Positions of REP sequences were taken from the Ecocyc database (http://ecocyc.org)^48^. Positions of H-NS and FIS binding sites were taken from Kahramanoglou *et al.* (2011)^49^. Positions of IHF binding sites, ChIP-Seq data for HupA and HupB binding, and the change in gene expression between the HU knockout and the wild-type, were all taken from Prieto *et al.* (2012)^50^. For analysis of the ChIP-Seq data, reads were aligned to the *E. coli* K-12 MG1655 genome (NC_000913.2) using BWA^51^. Alignment files were converted to the BED format using functions in the SAMtools^52^ and BEDtools^53^ suites. Coverage was calculated as the number of reads starting at each genomic position.

## Additional information

**How to cite this article:** Lal, A. *et al.* Genome scale patterns of supercoiling in a bacterial chromosome. *Nat. Commun.* 7:11055 doi: 10.1038/ncomms11055 (2016).

## Supplementary Material

Supplementary Information Supplementary Figures 1-10 and Supplementary Methods
