# Supplementary material for: Genome scale patterns of supercoiling in a bacterial chromosome
Source: Nat Commun. 2016 Mar 30;7:11055. doi: 10.1038/ncomms11055 (PMC4820846; doi:10.1038/ncomms11055)
Supplement: Supplementary Information — Supplementary Figures 1-10 and Supplementary Methods [file ncomms11055-s1.pdf]

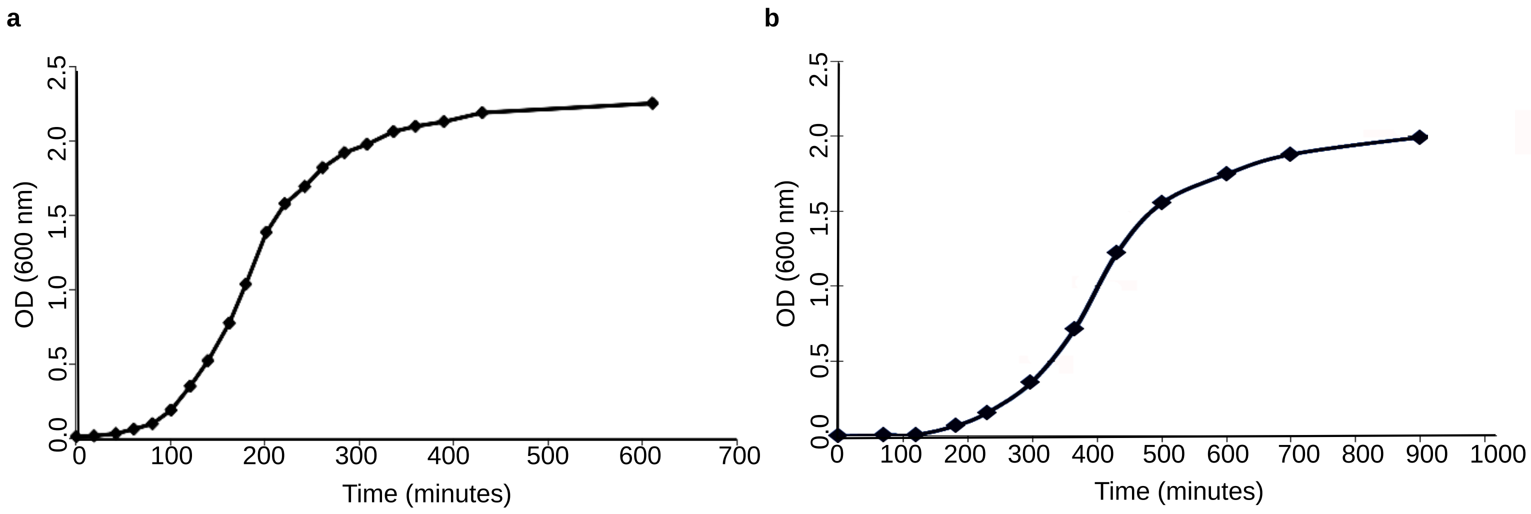

Supplementary Figure 1: Growth curves
Sample growth curves in LB medium for (a) Wild-type *E. coli* MG1655 and (b) Δ*hupAB.*

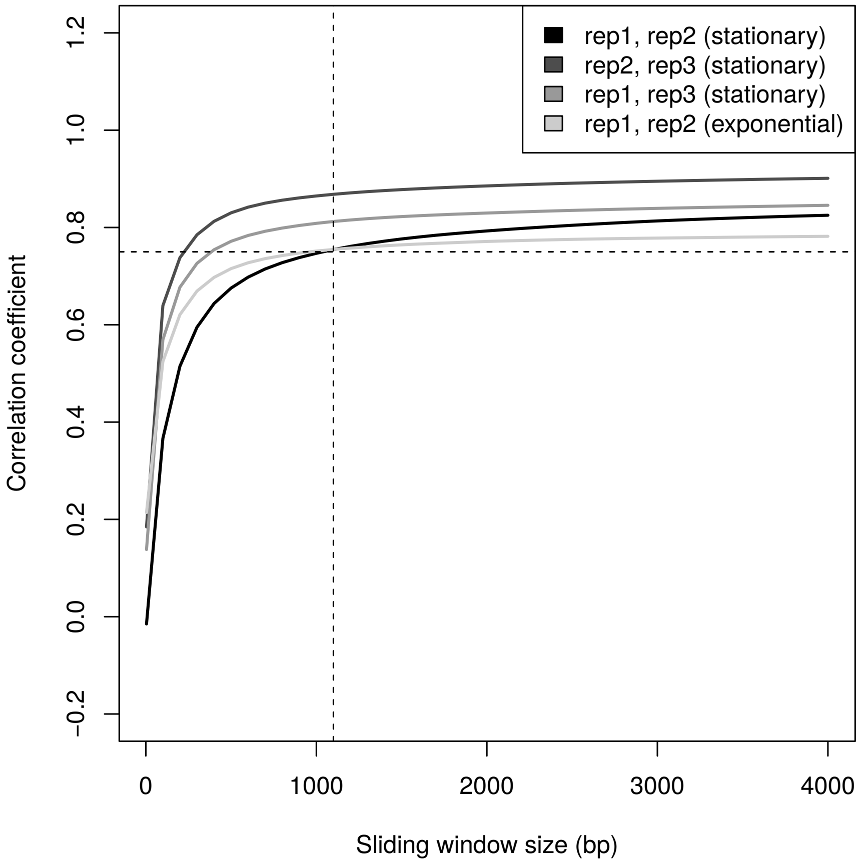

Supplementary Figure 2: Correlation between smoothed psoralen binding signals from replicate experiments.

The $\log_2$ ratio of the crosslinked and the non-crosslinked fluorescent signals corresponding to each probe on the microarray was calculated for three replicate experiments in stationary phase and two in exponential phase. The signals were then smoothed using a moving average with different window sizes. The y-axis shows the Pearson correlation coefficient between pairs of replicate experiments for different window sizes. To obtain correlations of 0.75 or higher between all replicates of the same experiment, a window size of 1.1 Kb was chosen.

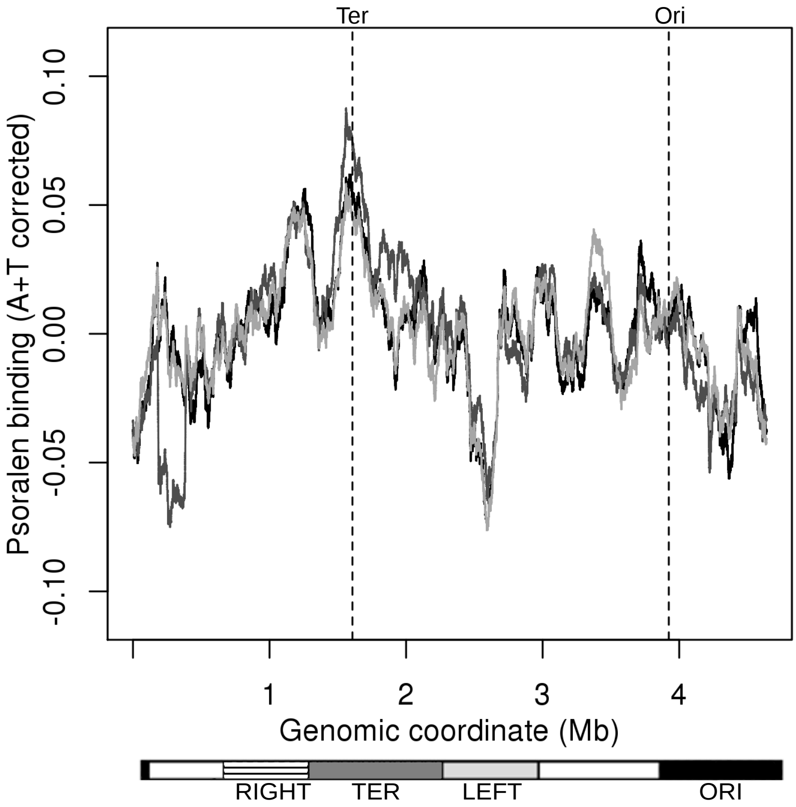

Supplementary Figure 3: Psoralen binding in stationary phase corrected for A+T content

In order to correct the psoralen binding signal for local A+T content, residuals of the loess fit between the $\log_2$(crosslinked/non-crosslinked) fluorescent signal and the A+T content of each microarray probe were taken for each of three replicate experiments (black, dark gray and light gray tracks) in stationary phase cells. The plot shows the moving average of the signal with a window size of 200 Kb. Dashed lines mark the terminus and the origin of replication. Lower bars represent chromosomal macrodomains.

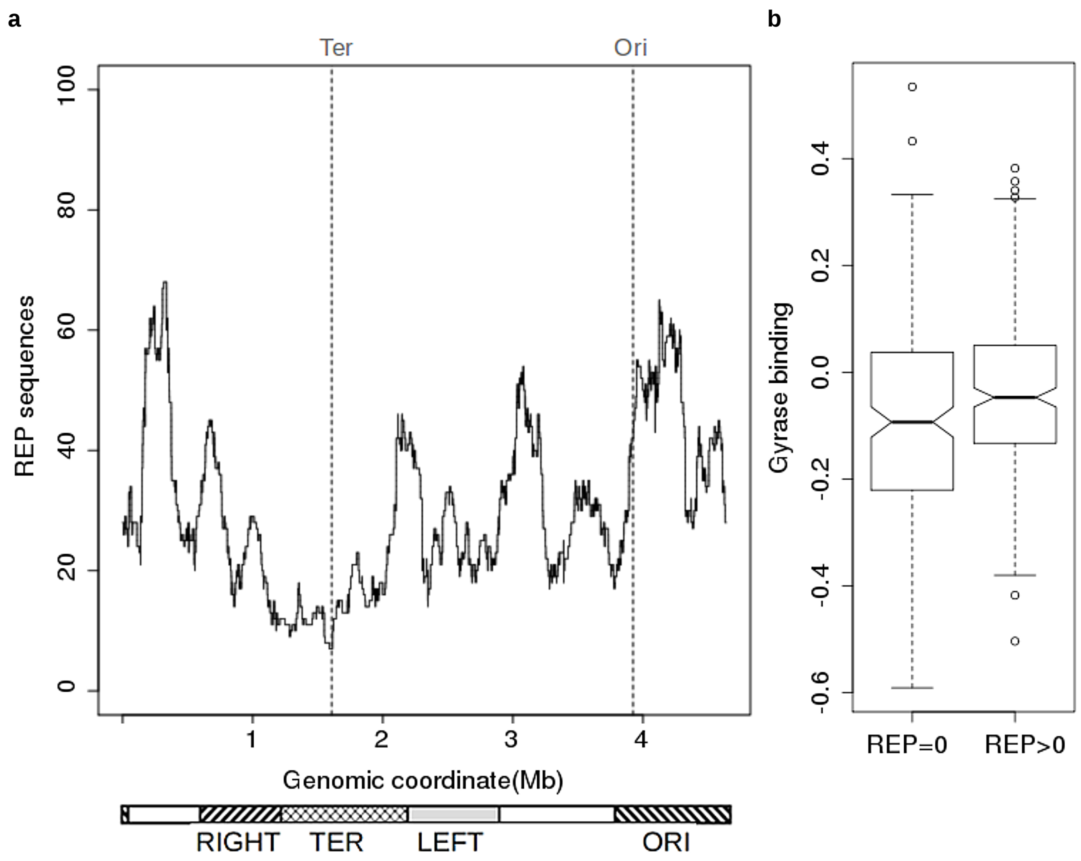

Supplementary Figure 4: REP sequences

(a) Distribution of REP sequences over the *E. coli* genome. The number of REP sequences in a 200 Kb sliding window is plotted. Dashed lines mark the terminus and origin of replication. Lower bars show locations of chromosomal macrodomains. (b) The *E. coli* genome was divided into 10 Kb bins. Boxplot shows DNA gyrase binding in genes within a bin containing at least one REP sequence and in bins without REP sequences.

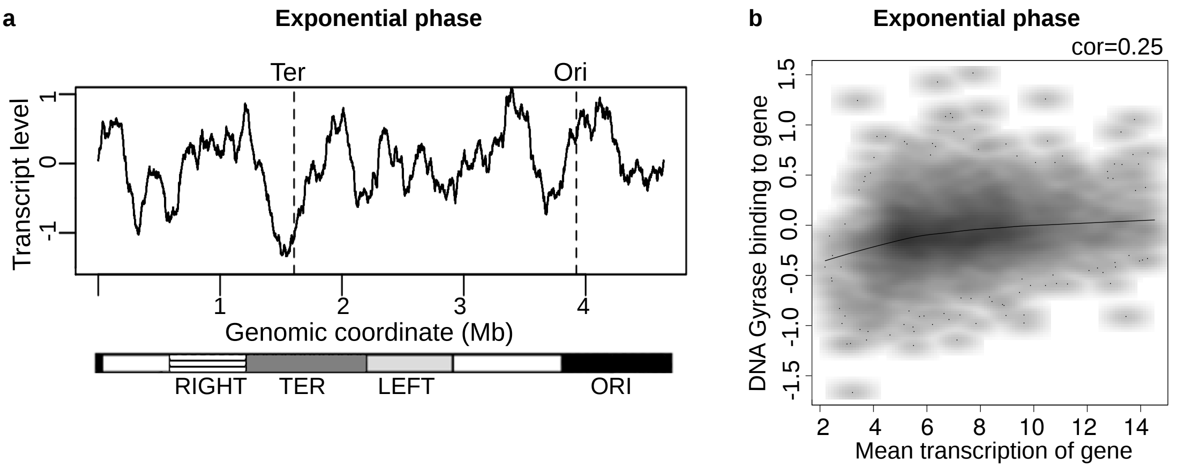

Supplementary Figure 5: Correlation between exponential phase transcription and DNA gyrase binding.

(a) cDNA derived from cellular RNA was hybridized to tiling arrays covering the *E. coli* genome. The moving average of the microarray signal is plotted with a window size of 200 Kb in exponential phase. Dashed lines mark the positions of the terminus and origin of replication. Lower bars represent chromosomal macrodomains. (b) Smoothed scatter plot of exponential phase DNA gyrase binding to genes versus level of transcription.

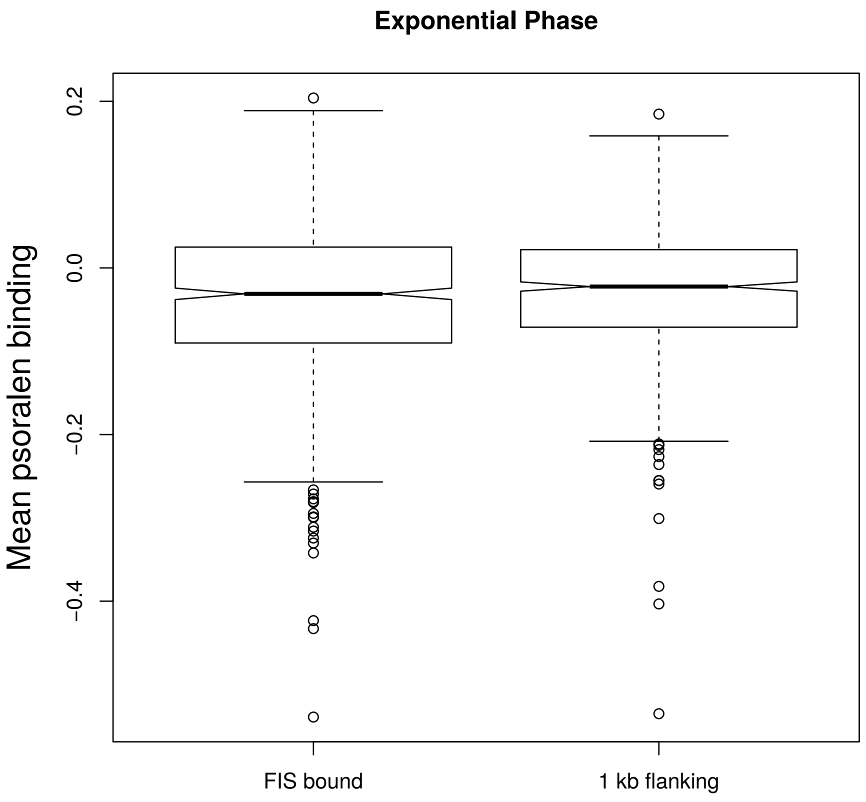

Supplementary Figure 6: Psoralen binding in FIS binding sites

Boxplot of average psoralen binding within FIS binding sites versus average psoralen binding in 1 Kb flanking regions during exponential phase.

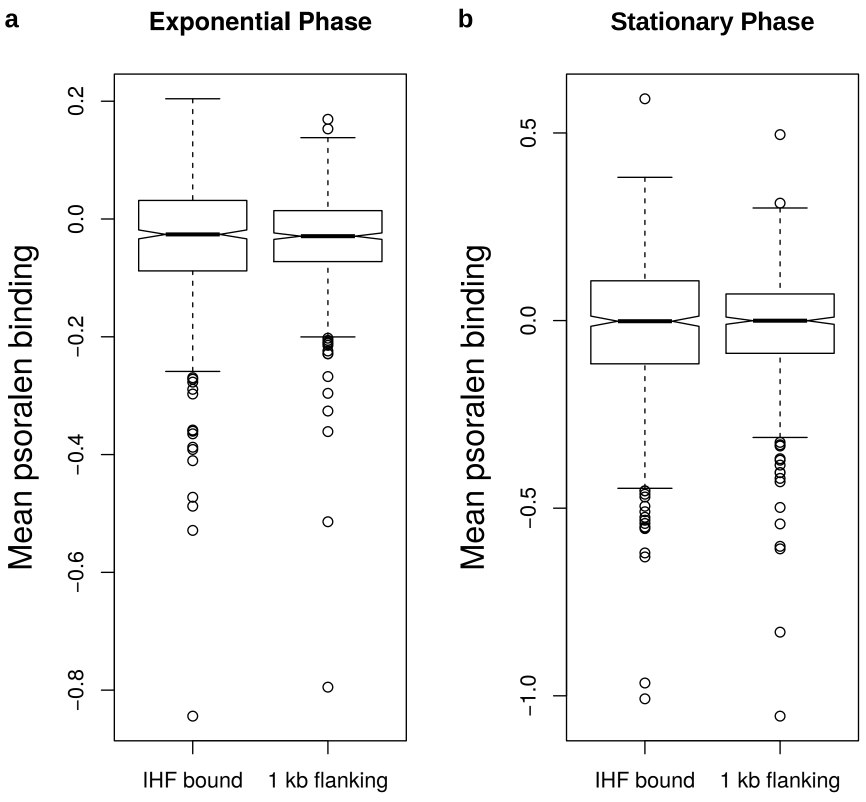

Supplementary Figure 7: Psoralen binding in IHF binding sites

Boxplot of average psoralen binding within IHF binding sites versus average psoralen binding in 1 Kb flanking regions during (a) exponential phase and (b) stationary phase.

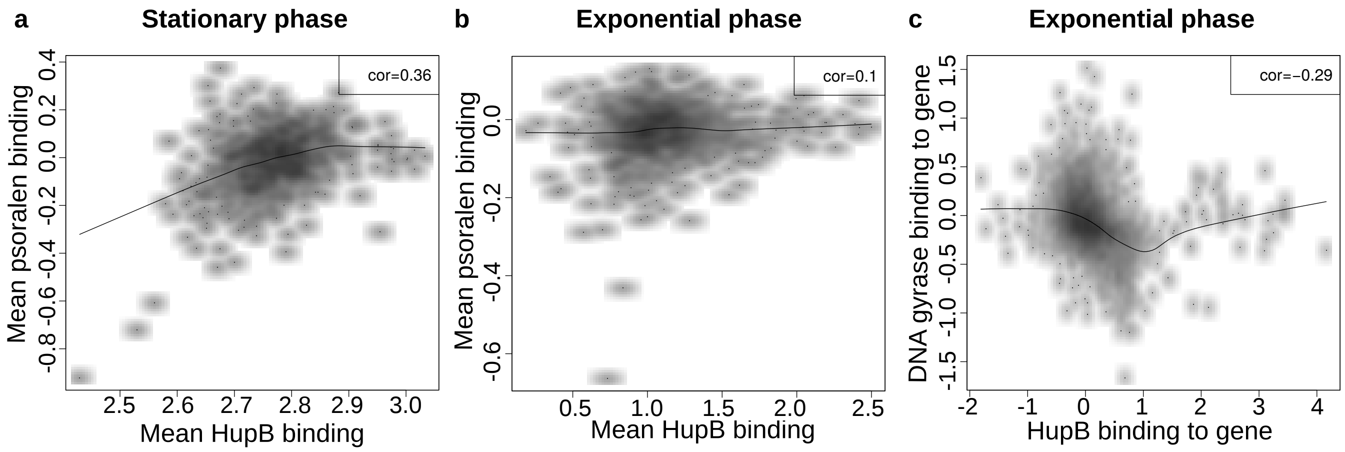

Supplementary Figure 8: Correlation between HU and psoralen binding using HupB ChIP-seq data

Smoothed scatter plot of psoralen binding versus HupB binding in (a) Stationary phase and (b) Exponential phase.
(c) Smoothed scatter plot of DNA gyrase binding versus average HupB binding within genes during exponential phase.
Black lines represent loess fit.

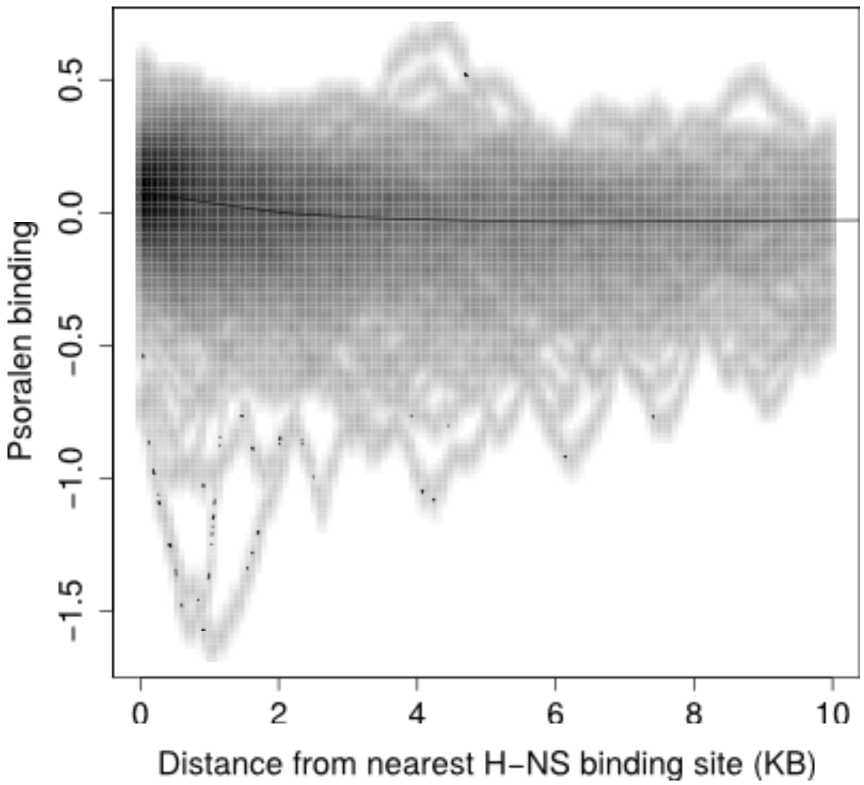

Supplementary Figure 9: Effect of H-NS on supercoiling decays with distance from its binding sites

Smoothed scatter plot of psoralen binding during stationary phase versus distance from the nearest end of an H-NS binding site, for positions on the genome outside H-NS binding sites. Black line represents the loess fit.

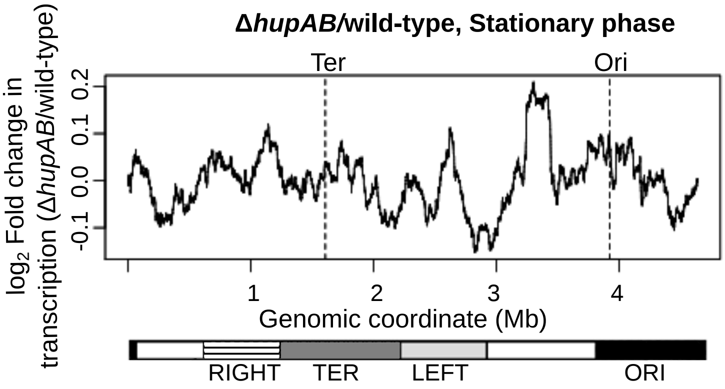

Supplementary Figure 10: Change in transcription in the HU knockout

$\log_2$ fold change in transcription between the HU knockout strain and the wild-type during stationary phase. The moving average is plotted with a window size of 200 Kb. Dashed lines mark the positions of the terminus and origin of replication. Lower bars show locations of chromosomal macrodomains.

Supplementary methods

E. coli transcriptome assay

Cells from 12.5 ml culture were harvested by centrifugation and RNA purified using the RNeasy® Mini Kit (Qiagen® 74104) following the manufacturer's recommendations. Further sample processing was performed according to the Affymetrix GeneChip® Expression Analysis Technical Manual, Section 3. Isolated RNAs (10 ug) were used for Random Primer cDNA synthesis using SuperScript II™ Reverse Transcriptase, (Invitrogen Life Technologies 18064-071). RNA was removed by treating the reaction mixture with 1N NaOH followed by neutralizing with 1N HCl. cDNAs were purified using MiniElute® PCR Purification columns (Qiagen® 28004) and fragmented to 50-200 bp by 0.6U/ug of DNase I (Amersham Biosciences 27-0514-01) for 10 minutes at 37°C in 1X One-Phor-All buffer (Amersham Biosciences 27-0901-02). DNase I was inactivated by heating at 98°C for 10 minutes. Fragmented cDNAs were then 3' termini biotin labeled using the GeneChip® DNA Labeling Reagent (Affymetrix 900542) and 60U of Terminal Deoxynucleotidyl Transferase (Promega M1875) at 37°C for 60 minutes. The labeling reaction was stopped by the addition of 0.5M EDTA. Labeled cDNA fragments (3ug) were then hybridized for 16 hours (60 rpms) at 45°C to tiling array chips (Ecoli_Tab520346F).
